# Single-Cell Transcriptomics Reveals Splicing Features of Adult Neural Stem Cells in the Subventricular Zone

**DOI:** 10.3389/fcell.2022.822934

**Published:** 2022-03-01

**Authors:** Yanlu Wang, Chun Li, Xi Gong, Xiao Chen, Chenming Liu, Hailei Zhang, Siguang Li, Yuping Luo

**Affiliations:** ^1^ Human Aging Research Institute and School of Life Science, Nanchang University, Nanchang, China; ^2^ Clinical and Translational Research Center, Shanghai First Maternity and Infant Hospital, Tongji University School of Medicine, Shanghai, China; ^3^ Key Laboratory of Spine and Spinal Cord Injury Repair and Regeneration of Ministry of Education, Orthopedic Department of Tongji Hospital, Tongji University School of Medicine, Shanghai, China; ^4^ College of Architectural Engineering, Jiangxi Science and Technology Normal University, Nanchang, China; ^5^ Novogene Bioinformatics Technology Co., Ltd., Beijing, China

**Keywords:** alternative splicing, single-cell transcriptomics, adult neural stem cells, bicistronic transcripts, subventricular zone

## Abstract

The central nervous system has enormously complex cellular diversity with hundreds of distinct cell types, yet alternative splicing features in single cells of important cell types at neurogenic regions are not well understood. By employing *in silico* analysis, we systematically identified 3,611 alternative splicing events from 1,908 genes in 28 single-cell transcriptomic data of adult mouse ependymal and subependymal regions, and found that single-cell RNA-seq has the advantage in uncovering rare splicing isoforms compared to bulk RNA-seq at the population level. We uncovered that the simultaneous presence of multiple isoforms from the same gene in a single cell is prevalent, and quiescent stem cells, activated stem cells, and neuroblast cells exhibit high heterogeneity of splicing variants. Furthermore, we also demonstrated the existence of novel bicistronic transcripts in quiescent stem cells.

## Introduction

Alternative splicing (AS) events of precursor mRNAs are widespread in eukaryotic organisms and enable cells to generate vast protein diversity from a limited number of genes (Gilbert, 1978; Pan et al., 2008; Barbosa-Morais et al., 2012). AS events detected in nervous system tissues are especially prevalent and more highly conserved than those detected in other tissues, suggesting that they more often provide essential functions (Jelen et al., 2007; Merkin et al., 2012; Raj and Blencowe, 2015). It is now established that AS events play diversified regulation roles such as neural cell differentiation, neuronal migration, synapse formation, and brain development in the central nervous system (CNS) (Yano et al., 2010; Grabowski, 2011; Zheng et al., 2012; Shin and Kim, 2013; Mauger et al., 2015). Disruptions in the splicing machinery can result in neurodegeneration (Polymenidou et al., 2011). These findings emphasize the importance of characterizing AS events in CNS tissues and understanding its spatiotemporal and dynamic regulations.

Previous population-based studies revealed characteristics and spatiotemporal regulation of AS in the CNS (Jelen et al., 2007; Yano et al., 2010; Grabowski, 2011; Zheng et al., 2012; Shin and Kim, 2013; Mauger et al., 2015; Tilgner et al., 2015; Suzuki et al., 2017; Jackson et al., 2018; Weyn-Vanhentenryck et al., 2018). The CNS has enormously complex cellular diversity with hundreds of distinct cell types, while population-based AS analyses of bulk tissues with heterogeneous cellular composition reveal the average of all of the different cells in a particular tissue and does not truly reflect functions of any particular or rare but important cell types in these tissues. To circumvent such a problem, single-cell-based alternative splicing analyses become imperative. By single-cell RNA sequencing, researchers identified cell-type-specific mRNA isoforms in the mouse primary motor cortex and cerebellum (Gupta et al., 2018; Booeshaghi et al., 2021) and mRNA isoform diversity of oligodendrocyte and vascular and leptomeningeal cells in the mouse brain (Karlsson and Linnarsson, 2017), and found splicing dynamics during the differentiation of human iPSCs to motor neurons or neuron progenitor cells (Song et al., 2017). At present, few research efforts have been dedicated to AS studies in a single cell at neurogenic regions.

The subventricular zone (SVZ) of the adult mouse forebrain, one of the known neurogenic regions, harbors neural stem cells (NSCs) that migrate to the olfactory bulb along the rostral migratory stream (RMS) and give rise to olfactory bulb interneurons throughout life (Altman, 1969; Doetsch et al., 1999). This region contains multiple distinct cell types related to adult NSC activities (Doetsch et al., 1999). The lack of the single-cell-level insight on isoform expression of ependymal and subependymal cells hampered the understanding of the biological role of alternative splicing in these regions. In this study, we analyzed single-cell transcriptomic data from adult mouse ependymal and subependymal cells, identified 3,611 AS events from 1,908 genes, and systematically characterized their detailed AS features. Moreover, we uncovered that genes expressing multiple splice isoforms simultaneously in a single cell are prevalent. Finally, we demonstrated the existence of new bicistronic transcripts in single quiescent stem cells.

## Results

### Landscape of AS Event Profiles in Single Cells of the Mouse SVZ Region

To identify AS events of individual cell types in the SVZ region, we performed an isoform identification pipeline to analyze transcriptomic data derived from 28 single-cell samples which we have obtained previously (Luo et al., 2015) from adult mouse CD133 positive and negative ependymal/subependymal cells. These single cells comprised quiescent neural stem cells, activated neural stem cells, neuronal lineage, oligodendrocytes, and other cells in the SVZ region (Luo et al., 2015). An average depth of 15–20 million reads per cell was obtained, and when an FPKM (fragments per kilobase of transcript per million mapped reads) cutoff at 0.1 was chosen, all samples reached saturation in mRNA detection with about 8 million reads used (Luo et al., 2015), suggesting sequencing depths of single-cell transcriptomics data were sufficient for AS analysis.

**FIGURE 1:**
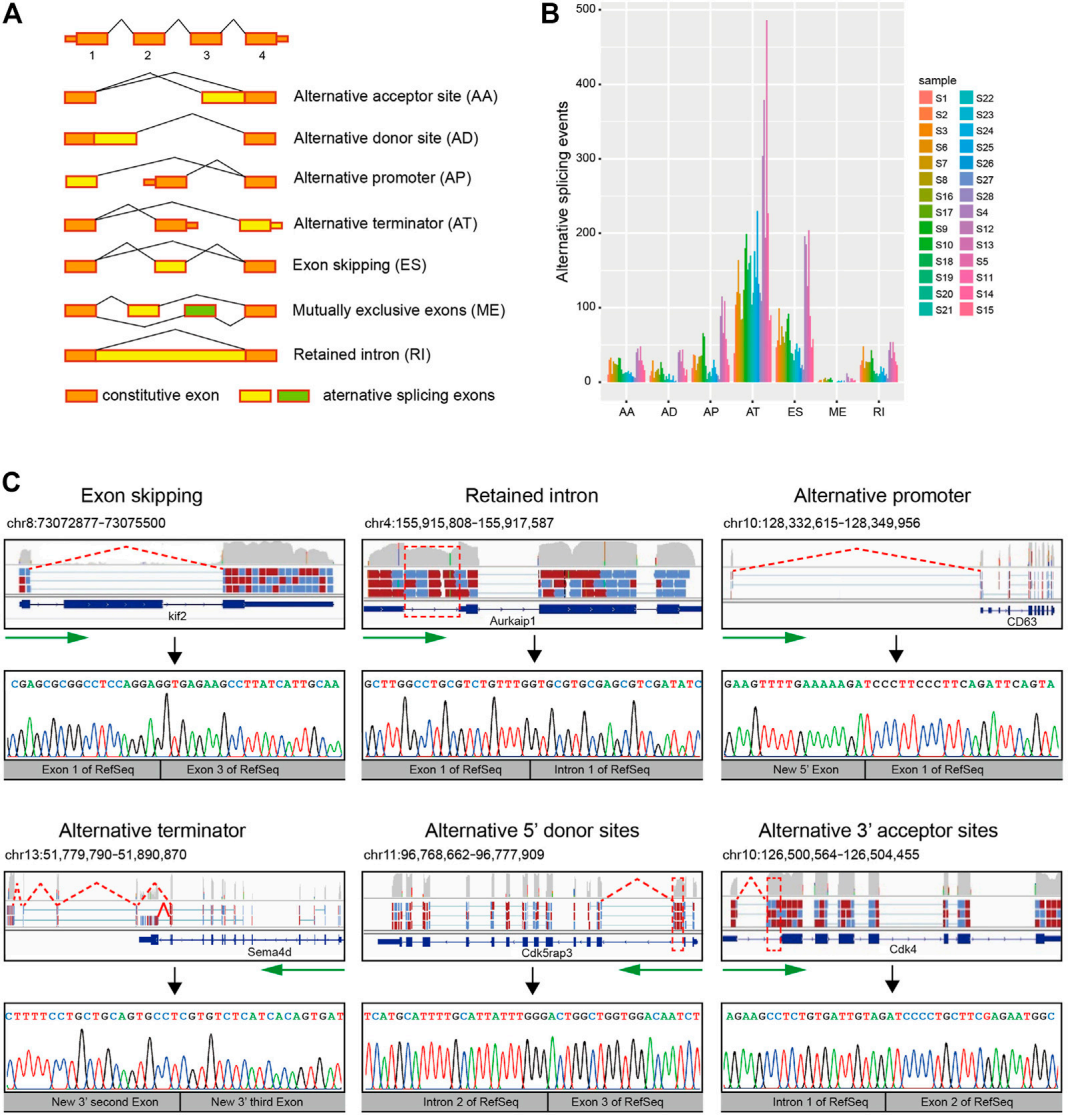
Detection of AS events in the mouse SVZ region by single-cell RNA-seq. **(A)** A diagrammatic sketch of the seven types of AS events identified in the present study. **(B)** Number and type of AS events for 28 single-cell samples. Samples were illustrated according to the cell types. Quiescent stem cells: S1, S2, S3, S6, S7, S8, S16, and S17. Activated stem cells: S9 and S10. Neuroblast cells: S18, S19, S20, S21, S22, S23, S24, S25, S26, S27, and S28. Oligodendrocyte: S4, S12, and S13. Other cells: S5, S11, S14, and S15. **(C)** Schematic diagram, visualization, and verification of six major categories of local splicing patterns. Schematic diagram and visualization of six major categories of local splicing patterns (top); arrows in green mark the direction of transcription, and the location of alternative splicing (boxed) are indicated. Verification of six major categories of local splicing patterns by Sanger sequencing (bottom).

By performing the AS analysis approach described in a previously published study (Ryan et al., 2012), we identified 3,611 AS events from 1908 genes in the 28 single-cell samples. All the 3,611 AS events identified belong to different categories (Figure 1A) of local splicing patterns : alternative 3′ acceptor sites (AA), alternative 5’ donor sites (AD), alternative promoter (AP), alternative terminator (AT), exon skipping (ES), mutually exclusive exons (ME), and retention intron (RI). The distribution of AS events among different single cells is different in which alternative terminator events are the most frequent, followed by exon skipping. Furthermore, the quantity of AS events is inconsistent among single cells. For example, there are 947 AS events in CD133-negative cell S5, while only 141 AS events in neuroblast cell S28 (Figure 1B; Supplementary Table S1).

To verify that these isoforms were not artifacts of the amplification process required for constructing single-cell libraries, we isolated RNA from bulk tissue of the adult subventricular zone of three-week-old mice and then performed RT-PCR with primers designed for randomly selected 10 AS events and Sanger sequencing for confirming these 10 AS events. Our results showed that all these detected AS events can be verified by Sanger sequencing (Figure 1C; Supplementary Figure S1), suggesting our single-cell RNA-seq is effective for alternative splicing analysis.

### Discovery of Low-Abundance Splicing Isoforms in Cells From the Subventricular Zone by Single-Cell RNA-Seq

**FIGURE 2:**
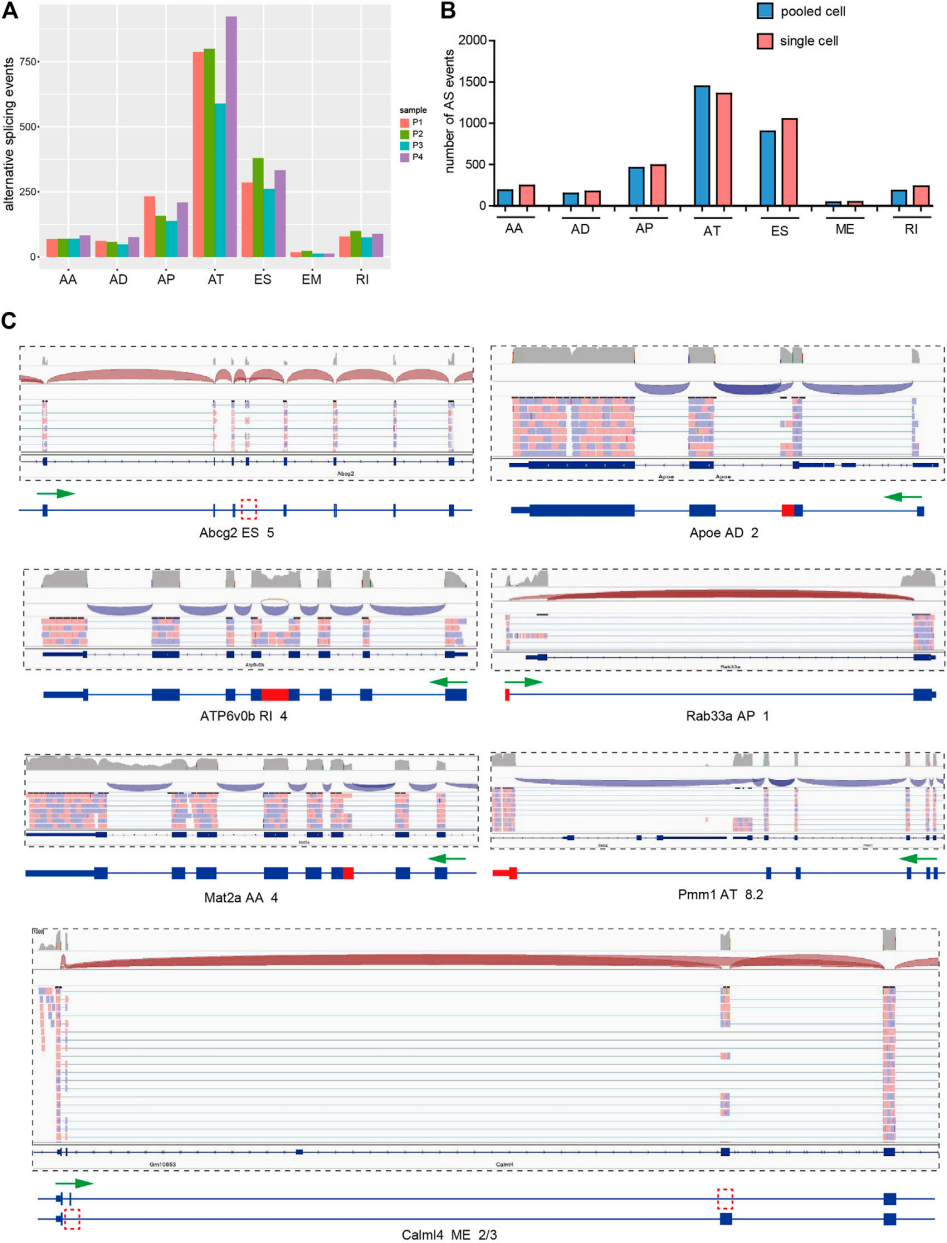
Discovery of low-abundance AS events by single-cell RNA-Seq. **(A)** Number and type of AS events of four pooled-cell samples. The pooled-cell samples were collected and mixed ten CD133-positive and ten CD133-negative single-cell libraries, respectively, and two mixed/pooled ten CD133-positive samples and two mixed/pooled ten CD133-negative samples were sequenced. **(B)** Comparison of number and type of AS events between 28 single-cell samples and 4 pooled-cell samples. **(C)** Example of low-abundance and novel AS events existing only in single-cell samples showed by IGV visualization. Arrows in green mark the direction of transcription, and the location of alternative splicing is indicated in red.

Population-based AS analyses of bulk tissues with heterogeneous cellular composition reveal the average of AS events from all of the different cells in that particular tissue. In order to know the differences of AS events in pooled-cell and single-cell levels, we analyzed AS events in 4 pooled-cell samples with mixed ten single-cell libraries in each sample (Figure 2A) and compared the variance of different types and the quantity of AS events between 28 single-cell samples and 4 pooled-cell samples. We found that there were no significant differences in the proportion of AS types detected between the single and pooled cells (Figure 2B). However, more AS events were detected in 28 single-cell samples (3,611) than in 4 ten-cell pooled samples (3,379 in 40 cells). Notably, differentially expressed alternative splicing (DEAS) analysis showed that most of DEAS events detected only in single-cell samples are low-abundance and novel splicing isoforms (Figure 2C), suggesting single-cell AS analyses could find low-abundance AS events present in rare cell types and could not find in population-based AS analyses.

### Detection of Highly Heterogenetic Splice Variants in Quiescent Stem Cells, Activated Stem Cells, and Neuroblast Cells

**FIGURE 3:**
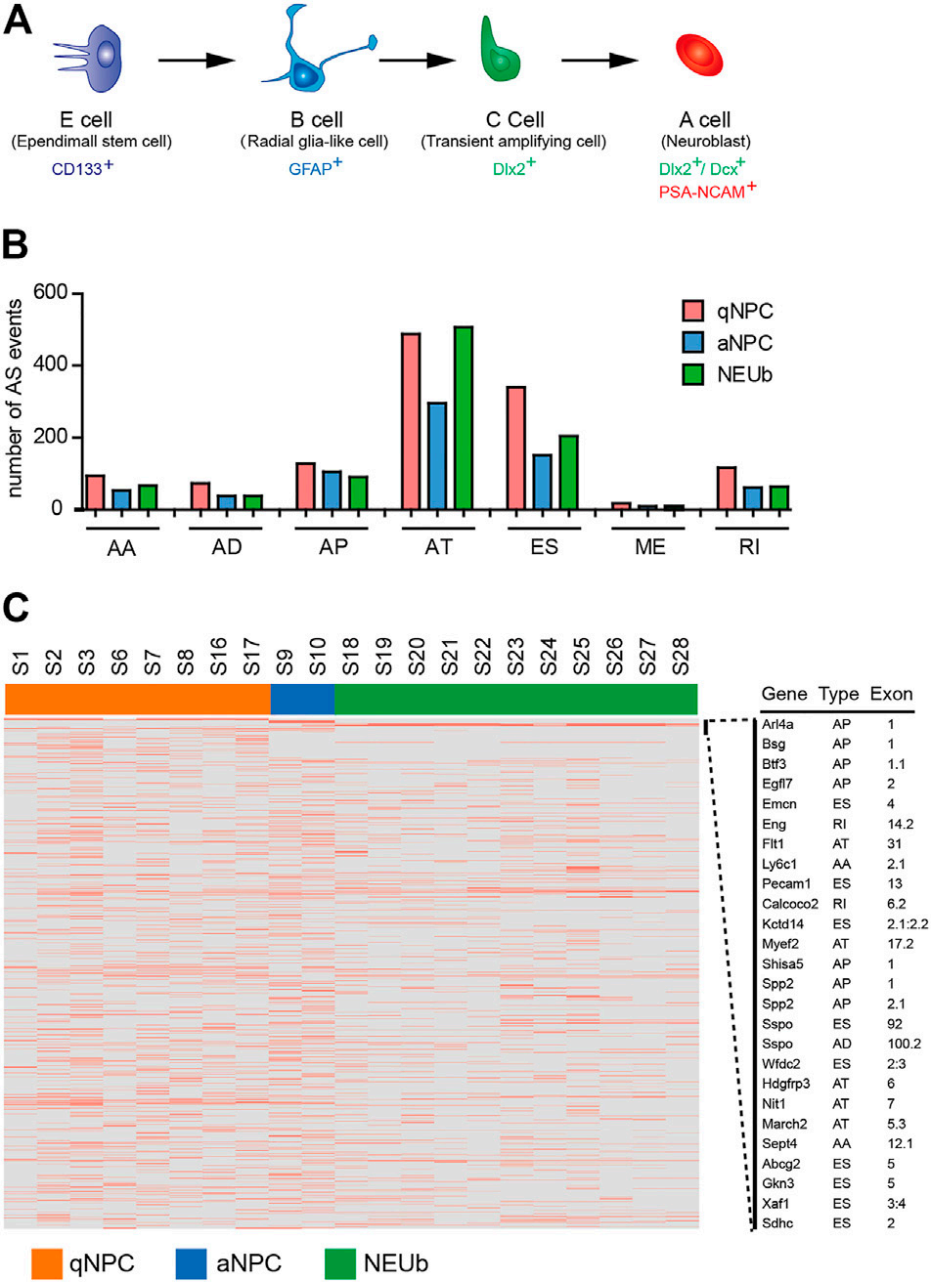
Detection of highly heterogenetic splice variants in stem cells of the SVZ. **(A)** Schematic diagram of major cell types in the adult mouse subventricular/subependymal zone (SVZ/SEZ). CD133^+^ E cells represent some quiescent stem cells; GFAP^+^ B cells are also regarded as SEZ-activated neural stem cells (NSCs); C cells are the transitional cells from B to A cells, and A cells are neuroblasts. **(B)** Comparison of number and type of AS events among quiescent neural stem cells, activated neural stem cells, and neuroblast cells. **(C)** Heatmap of cell-type-specific and differentially expressed alternative splicing events. The horizontal axis shows the clustering information of samples divided into three major clusters: quiescent neural stem cells (8 samples), activated neural stem cells (2 samples), and neuroblast cells (11 samples).

The ependymal/subependymal region contains the previously described four cell types related to adult NSC activities: ependymal E cells, subependymal B cells, transit-amplifying C cells, and neuroblast A cells (Figure 3A). It has been reported that CD133^+^ ependymal quiescent NSCs in ependymal E cells can be mitotically activated (B cells) and give rise to neuroblast A cells in the rostral migratory stream (RMS) and interneurons in the olfactory bulb (Doetsch, 2003; Coskun et al., 2008; Luo et al., 2015). It is reasonable to consider that differentially expressed AS events (DEAS) among quiescent NSCs, activated NSCs, and neuroblast cells may be relevant to the activation and differentiation of quiescent NSCs. To identify the splice variants during the activation and differentiation of quiescent NSCs, we analyzed single-cell RNA-seq data of 8 CD133^+^ ependymal quiescent NSCs, 2 activated NSCs, and 11 neuroblast cells from the SVZ region (Luo et al., 2015). Results showed that the proportion of AS types was consistent among quiescent NSCs, activated NSCs, and neuroblast cells (Figure 3B). However, of the 1,256 AS events detected in quiescent NSCs, 712 AS events were in activated NSCs, and 977 AS events were in neuroblast cells. Only a few AS events were cell-type-specific, and most of them were highly heterogeneous (Figure 3C; Supplementary Figure S2). For example, the SPP2 gene is expressed only in activated NSCs and showed the presence of activated NSC-specific alternative promoters (Supplementary Figure S2A). However, the Esam gene was only expressed in CD133^+^ ependymal quiescent NSCs, but it exhibited different isoforms in these cells. In addition, the details of the Esam isoform shared in the RefSeq database showed that it is a novel isoform with exon skipping (Supplementary Figure S2B). The Ddah2 gene showed three different isoforms in different individual neuroblast cells, respectively (Supplementary Figure S2C). We also found novel isoforms in Gkn3 and Sdhc, which existed only in one of the quiescent NSCs, respectively (Supplementary Figure S2D). These findings suggest that AS event analyses based on single-cell RNA-seq revealed a high heterogeneity of gene expression and isoform usage in quiescent stem cells, activated stem cells, and neuroblast cells (Figure 3C).

### Simultaneous Presence of Multiple Isoforms From the Same Gene in a Single Cell Is Prevalent

**FIGURE 4:**
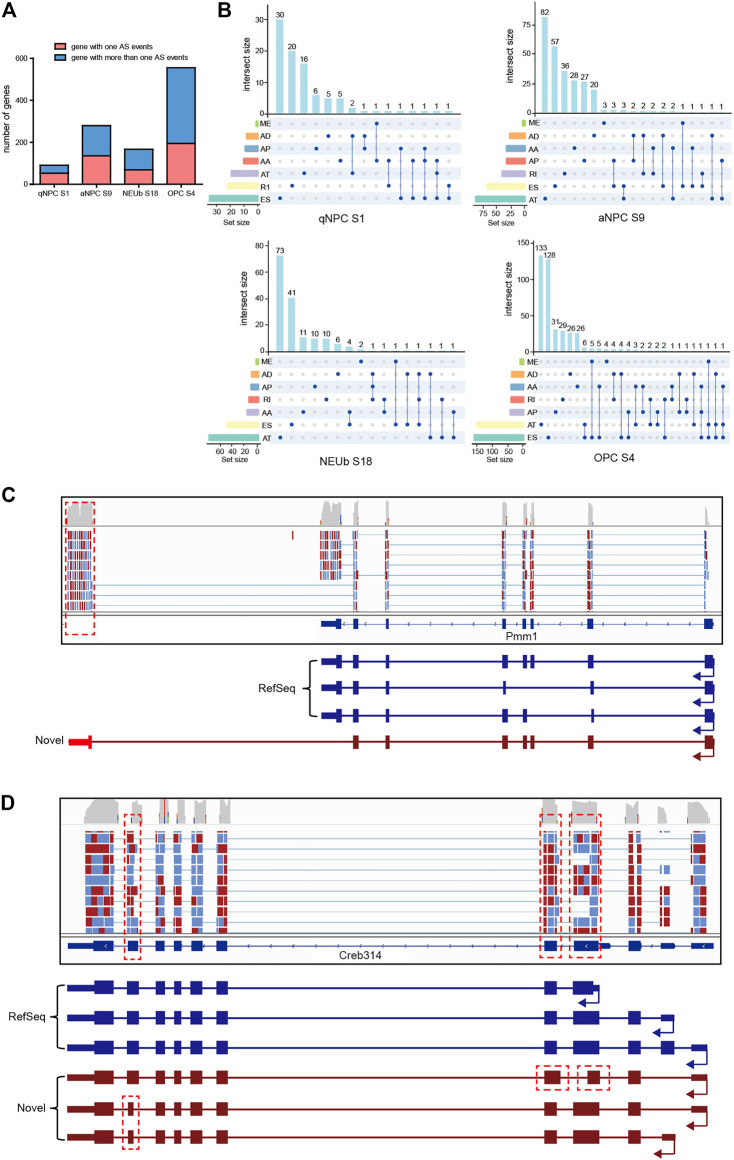
Isoform number analysis for a definite gene in individual cells. **(A)** Number of genes with one AS event and more than one AS event in four selected single cells for isoform number analysis. **(B)** One gene may incur more than one type of alternative splicing events in a single cell. **(C)** In an individual oligodendrocyte, the Pmm1 gene expressed two splice isoforms. **(D)** In an individual activated stem cell, the Creb3l4 gene expressed two splice isoforms. Isoforms in RefSeq are in blue, novel isoforms detected in this work are in brown, and the locations of novel alternative splicing events are indicated in red.

A previous study showed that for genes having multiple splice isoforms at the population level, individual cells predominantly expressed one particular isoform (Shalek et al., 2013). However, when Karlsson and Linnarsson sequenced six cDNAs stored in an earlier single-cell experiment, they found isoform diversity of single-cell mRNAs in oligodendrocytes (Karlsson and Linnarsson, 2017). To investigate the diversity of single-cell mRNA isoforms in the SVZ, we analyzed AS events and the corresponding parent genes of four samples, which were randomly selected from four cell types, including quiescent NSCs, activated NSCs, neuroblast cells, and oligodendrocyte cells, respectively. In the quiescent NSC sample 1 (S1), we identified 148 AS events from 91 genes, and nearly half of these genes expressed more than one isoform. In the activated NSC S9, we identified 438 AS events from 279 genes, and more than half of these genes expressed multiple isoforms. In neuroblast cell S18, we identified 275 AS events from 167 genes, and nearly two-thirds of these genes expressed more than one isoform. In oligodendrocyte cell S4, we identified 950 AS events from 545 genes, and more than two-thirds of the genes expressed more than one isoform (Figure 4A). Moreover, most of the parent genes occurred in only one type of AS event, whereas certain parent genes contained more than one type of AS event (Figure 4B). These statistical findings suggest that some genes can simultaneously express more than one particular isoform in a single cell. For example, Pmm1 and Creb3l4 genes can express multiple isoforms in an individual cell, some of which are novel splice isoforms, including the alternative terminator (Figure 4C), alternative 5′ donor sites, and alternative 3’ acceptor sites (Figure 4D). Coding potential analysis showed that the novel isoforms of the two genes possess protein-coding potential and can be expected to give rise to distinct protein isoforms similar to the original one.

To verify that simultaneous presence of multiple isoforms from the same gene in a single cell is the true biological property of these cells, rather than technical noise associated with the single-cell contamination, we analyzed our single-cell transcriptomics data of two-cell-stage mouse developing embryos, where the two sister cells are easily isolated and avoid cases of more than one cell, and we acquired consistent results as mentioned previously (Supplementary Figure S3). Together, our results reveal that the simultaneous presence of multiple isoforms from the same gene in a single cell is prevalent, and single-cell isoform diversity is a widespread event, which affects the protein-coding repertoire.

### Discovery of Bicistronic Transcripts in Quiescent Stem Cells

We detected new transcriptional fusions of adjacent genes in single cells of SVZ, including a fusion of the Hyal1 and Nat6 mRNA (Supplementary Figure S1A), a fusion of the Ramp2 and Vps25 mRNA (Supplementary Figure S1C), a fusion of the Nme1 and Nme2 mRNA (Supplementary Figure S1E), and a fusion of the Gimap1 and Gimap5 mRNA (Supplementary Figure S1F). A fused transcript was formed by transcription of two consecutive genes, in which partial 5′ sequence of upstream mRNA and partial 3’ sequence of downstream mRNA were fused into a single transcript (for most cases, there is a strong tendency to lose the last and first exons of the upstream and downstream mRNA, respectively). By RT-PCR, cloning, and Sanger sequencing, we verified the presence of the first two fused transcripts (Supplementary Figures S1B, D). Among the fused mRNAs mentioned previously, Nme1-Nme2 and Gimap1-Gimap5 are homologs of human fused mRNAs (Akiva et al., 2006; Parra et al., 2006), suggesting that fused transcripts are evolutionarily conserved.

**FIGURE 5:**
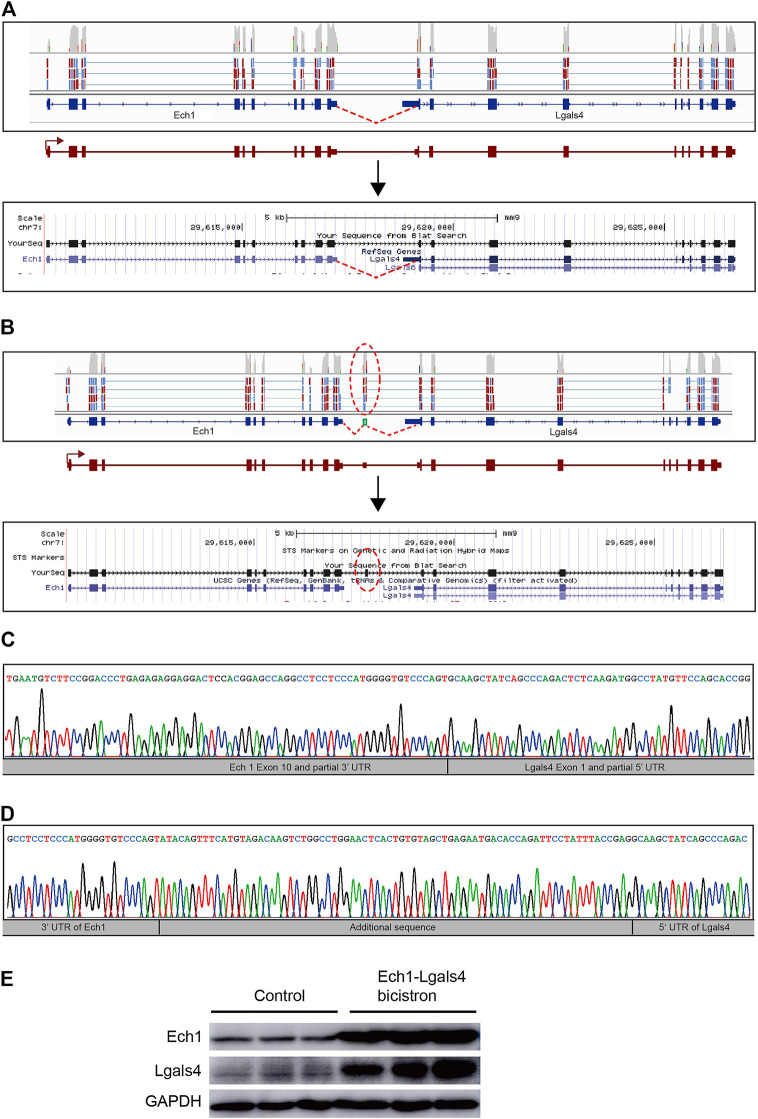
Discovery of bicistronic transcripts in quiescent NSCs. **(A)** Visualization of one of the bicistronic Ech1–Lgals4 transcripts in quiescent NSCs performed by using an Integrated Genomic Viewer (top) and a UCSC Genome Browser (bottom). Novel splice-junction spanning reads were detected from partial 3′UTR of the Ech1 transcript into partial 5′UTR of the Lgals4 transcript (red lines) in RNA from a single cell in quiescent NSC. **(B)** Visualization of the other bicistronic Ech1-Lgals4 transcript in quiescent NSCs by using an Integrated Genomic Viewer (top) and a UCSC Genome Browser (bottom). Novel splice-junction spanning reads were detected from partial 3′UTR of the Ech1 transcript into a 77 bp 5′ of intergenic sequence between Ech1 and Lgals4 transcripts and from a 77 bp 3′ of intergenic sequences between Ech1 and Lgals4 transcripts into partial 5′UTR of the Lgals4 transcript (red ellipses) in RNA from a single cell in quiescent NSCs. **(C)** and **(D)** Sanger sequencing of reverse transcriptase polymerase chain reaction products from mouse SVZ samples confirms the existence of bicistronic transcripts illustrated in **(A)** and **(B)**, respectively. **(E)** Western blot of Ech1 and Lgals4 protein expression in HEK293T transfected with the bicistronic Ech1–Lgals4 transcript constructs.

Interestingly, in the analysis of tandem gene fusion events, we detected novel bicistronic transcripts, where two adjacent genes (Ech1 and Lgals4) in the same orientation were transcribed into a single RNA sequence that retains the ORF of the two original genes (Figure 5). There were two different types of bicistronic Ech1–Lgals4 transcripts in two different single quiescent stem cells, respectively. In one bicistronic transcript, the ORFs of Ech1 and Lgals4 were linked directly by truncated 3′UTR of Ech1 and truncated 5′UTR of Lgals4 (Figure 5A). In the other bicistronic transcript, the ORFs of Ech1 and Lgals4 were linked by 77 nucleotides originating from the intergenic regions of Ech1 and Lgals4 (Figure 5B). To experimentally test the novel bicistronic transcripts, we isolated total RNA from the mouse SVZ and used it as a template for RNA reverse transcription reaction. By RT-PCR, cloning, and Sanger sequencing, we verified the presence of the two novel bicistronic transcripts (Figures 5C,D).

The fused transcript can lead to a new, fused protein that has the properties of two original proteins (Thomson et al., 2000; Pradet-Balade et al., 2002). Unlike fused transcripts, bicistronic transcripts retain the ORFs of the two original genes and can be translated into two proteins (Gray et al., 1999; Schaefer and Brösamle, 2009). To determine the expression levels of Ech1 and Lgals4 at the translation level, we cloned two different types of bicistronic Ech1-Lgals4 transcripts (with or without 77 nucleotides originated from the intergenic region of Ech1 and Lgals4) into pCMV-N-HA, respectively. We transfected the two recombinant pCMV-N-HAs into 293T cells, respectively, and use the empty pCMV-N-HA as control. We then examined the expression levels of Ech1 and Lgals4 in control cells and bicistronic transcript overexpressed 293T cells. We found that Ech1 and Lgals4 protein levels were higher only in the cells that overexpressed the bicistronic transcript with 77 nucleotides than in the control group (Figure 5E). These results suggest that the sequences between the ORF of Ech1 and Lgals4 in this bicistronic transcript can drive the expression of Lgals4.

## Discussion

Alternative splicing of pre-messenger RNA is an essential mechanism for generating multiple transcripts and increasing the proteome complexity. Although AS events have been analyzed in CNS tissue and purified cell types in the brain (Zhang et al., 2014; Karlsson and Linnarsson, 2017; Song et al., 2017; Gupta et al., 2018; Weyn-Vanhentenryck et al., 2018) by bulk or single-cell analyses, the AS features in the adult mouse forebrain neurogenic zones remained unclear. In this study, we identified 3,611 AS events from 1,908 genes in the subventricular zone by systematically analyzing 28 single-cell transcriptomics data, which provided an initial picture of the AS patterns in ependymal quiescent, activated neural stem cells, and other cells in close proximity.

To date, several studies demonstrated that single-cell RNA-seq has provided valuable insights into cell-type-specific AS events (Li et al., 2015; Zhang et al., 2016; Karlsson and Linnarsson, 2017; Gupta et al., 2018; Booeshaghi et al., 2021). However, our single-cell AS analysis showed that most AS events were not cell-type-specific except for a few cases and revealed tremendous AS event varieties in ependymal quiescent neural stem cells, activated neural stem cells, and neuroblast cells (Figure 3). This splicing heterogeneity is largely due to cellular diversity, which may have resulted from programmed specialization during the activation of quiescent neural stem cells and differentiation of activated neural stem cells, or through random processes that occur in cells. Although most novel isoforms possess the protein-coding potential (Figure 3) with original open reading frames or new open reading frames, little is known about the significance of such variations and how many of the novel isoforms have biological functions or provide material for evolution.

In the present study, we found that single-cell isoform diversity is a widespread event. Of the 1,082 genes encoding 1,812 splicing isoforms in four selected single-cell samples for isoform number analysis and isoform diversity analysis (Figure 4), 639 (59%) of them expressed multiple isoforms simultaneously in a single cell. Those isoforms retain protein-coding potential, or code-truncated proteins, and/or non-coding RNAs. Thus, in a single cell, some genes can be expected to give rise to multiple distinct protein isoforms, greatly expanding the coding repertoire, or regulating gene expression through alternative stop codons, and non-sense-mediated decay (Lareau et al., 2007) or non-coding RNAs, suggesting that single-cell isoform diversity has a profound consequence on the fate of RNA molecules.

We have detected bicistronic transcripts in quiescent neural stem cells by single-cell RNA-seq and provided experimental evidence that these bicistronic transcripts do exist and are not mere technical artifacts. Although polycistronic operons are present in prokaryotes, bicistronic transcripts are rare in eukaryotes. Bicistronic transcripts have occasionally been reported in eukaryotes, and some evidence shows that two independent proteins can be translated from bicistronic transcripts in humans, mice, and zebrafish (Gray et al., 1999; Schaefer and Brösamle, 2009). However, the function of this transcript, as well as the mechanism involved in its generation and the actual impact of this phenomenon on the eukaryotic genomes, remains to be further elucidated.

## Materials and Methods

### Dataset Used in this Study

Single-cell and pooled-cell transcriptomic dataset (GSE61288) from Gene Expression Omnibus (GEO) was used for alternative splicing analysis. This dataset was prepared previously (Luo et al., 2015) from 12 adult mouse ependymal regions which include ependymal/subependymal quiescent NSCs, activated NSCs, and other cells in close proximity. This dataset was obtained using single-cell RNA sequencing technology (Tang et al., 2009, 2010) and can be used for alternative splicing analyses.

### Detection of Isoforms

Alternative splicing events of individual cell types in ependymal/subependymal regions were analyzed by SpliceSeq (Ryan et al., 2012). Then, the splicing isoforms were visualized using Integrated Genomic Viewer (IGV) and followed by consulting literature, comparing with NCBI Ref-seq, Ensembl 37.61, and UCSC Known Gene 4, and then, AS events from the single cell of adult mouse ependymal/subependymal regions were identified.

### Characterization of Alternative Splicing Events by PCR and Sanger Sequencing

To verify novel alternative splicing events in mouse different neuroanatomical regions and different tissues, three-week-old mice were anesthetized and euthanized in accordance with institutional guidelines of the Guide for Care and Use of Laboratory Animals (at Tongji University), and the brains and other tissues (including heart, liver, spleen, lung, kidney, stomach, large intestine, and small intestine) were isolated, and then be quickly put into cold PBS. The subventricular zone, hippocampus, olfactory bulbs, cortex, midbrain, and cerebellum were dissected from the brains under a dissection microscope. The total RNAs of different neuroanatomical regions of the brain and different tissues were extracted using RNAiso Plus (Takara Cat. # 9109). After the removal of contaminating DNA using RNase-free DNase (Ambion), 4 μg of total RNA was reverse-transcribed using RT Master Mix and according to manufacturer recommendations (Takara Cat. # RR036A).

PCR reactions were performed using 2 μl of cDNA with the EX Taq HS kit (Takara) and specified primers Klf2 (F: TAT​CTT​GCC​GTC​CTT​TGC​CA; R:TATCCCCAGCCACACCACTA); Aurkaip1 (F: GTT​GCG​GGG​CTC​AGA​TGA​T; R:CCAGGAACATGGTGAGCAGT); Cd63 (F: CAA​CCT​CGA​ACC​CGA​GTA​CC; R:TTCATTCCTCCTTCCACCGC); Sema4d (F:ACGTAGCAAGTTCCTGGCTC; R:GACTGTGTGTTAGCACGGGA); Cdk5rap3 (F1:ATCTTGTCAGCCCCAGCATC; R1:GGAGGCCATTGACCTGTACC; F2: GCTGGGCCGGGGTCTTT; R2:GTCCACCAGCCAGTCCCAAATAAT); Cdk4 (F:AAGTGTGGAGGGGTGGGAT; R:TTAATGGTCTCAACCGGCAG); Ech-Lgals4 (F:CGGGATCCCGCAGAAATGGCTACCGCGATG; R:GCTCTAGAGCCGGGGATCTTTCTGCTTCCT); Hyal1-Nat6 (:CGG​GAT​CCC​GTC​TAA​GCT​GGT​GCA​ACT​AGG​AC; R:CCCTCGAGGGACACAGGGTAAGGTTGTCCAC); and Ramp2-Vps25 (F:GGGGTACCCCCTCCTCGCCATCTCACCCAAG; R:GCTCTAGAGCGGGAGGTAAGAAGTAAAGGAGCC) (Invitrogen), according to the manufacturers’ recommendations. PCR products were separated on 1.5% agarose gel and then excised from the gel using the Qiagen DNA Gel Extraction Kit (Qiagen) according to the manufacturers’ recommendations. These DNA fragments were cloned to the pMD19-T vector (Takara) and then transformed to TOP10 chemically competent cells. The positive clones were selected by colony PCR and were submitted for Sanger sequencing.

### Western Blot Analysis

To test the expression of the novel bicistronic transcript Ech1-LGALS4, this bicistronic transcript was PCR-amplified from the SVZ and cloned into pCMV-N-HA (with primer F:CGGAATTCCGAGAAATGGCTACCGCGATGAR: GCT​CTA​GAG​CGG​AGC​TTA​GAT​GGA​ACT​CGG​G). Bicistronic transcript expression plasmids were transfected into HEK293 (BNCC No.100530), and the protein expression levels were analyzed using Western blot. For use, 30 µg protein samples were separated on SDS-PAGE gels and then transferred to PVDF membranes (BIO-RAD). The membranes were processed following the Western blotting protocol. Anti-ECH1 (Proteintech, No.11385-1-AP), anti-galectin-4 (Proteintech, No. 27552-1-AP), and anti-GAPDH (Abcam) were used as primary antibodies at the concentrations recommended by the manufacturers. HRP-conjugated secondary antibodies were obtained from Proteintech (No.SA00001-1, No. SA00001-2).

### Coding Potential Analysis of Novel Alternative Splicing Isoforms

The coding potential of novel alternative splicing isoforms was analyzed by using a coding potential calculator (CPC) (Kang et al., 2017), which is an SVM-based classifier comprehensively scoring the characteristics of a transcript including the presence and integrity of the predicted ORF, conservation of a single frame.

## Data Availability

Publicly available datasets were analyzed in this study. These data can be found here: Gene Expression Omnibus (GEO) (GSE61288).
