# Supplementary material for: Single-Cell Transcriptomics Reveals Splicing Features of Adult Neural Stem Cells in the Subventricular Zone
Source: Front Cell Dev Biol. 2022 Mar 1;10:822934. doi: 10.3389/fcell.2022.822934 (PMC8921602; doi:10.3389/fcell.2022.822934)
Supplement: Supplementary file 1 [file Presentation1.PPT]

## Slide 1
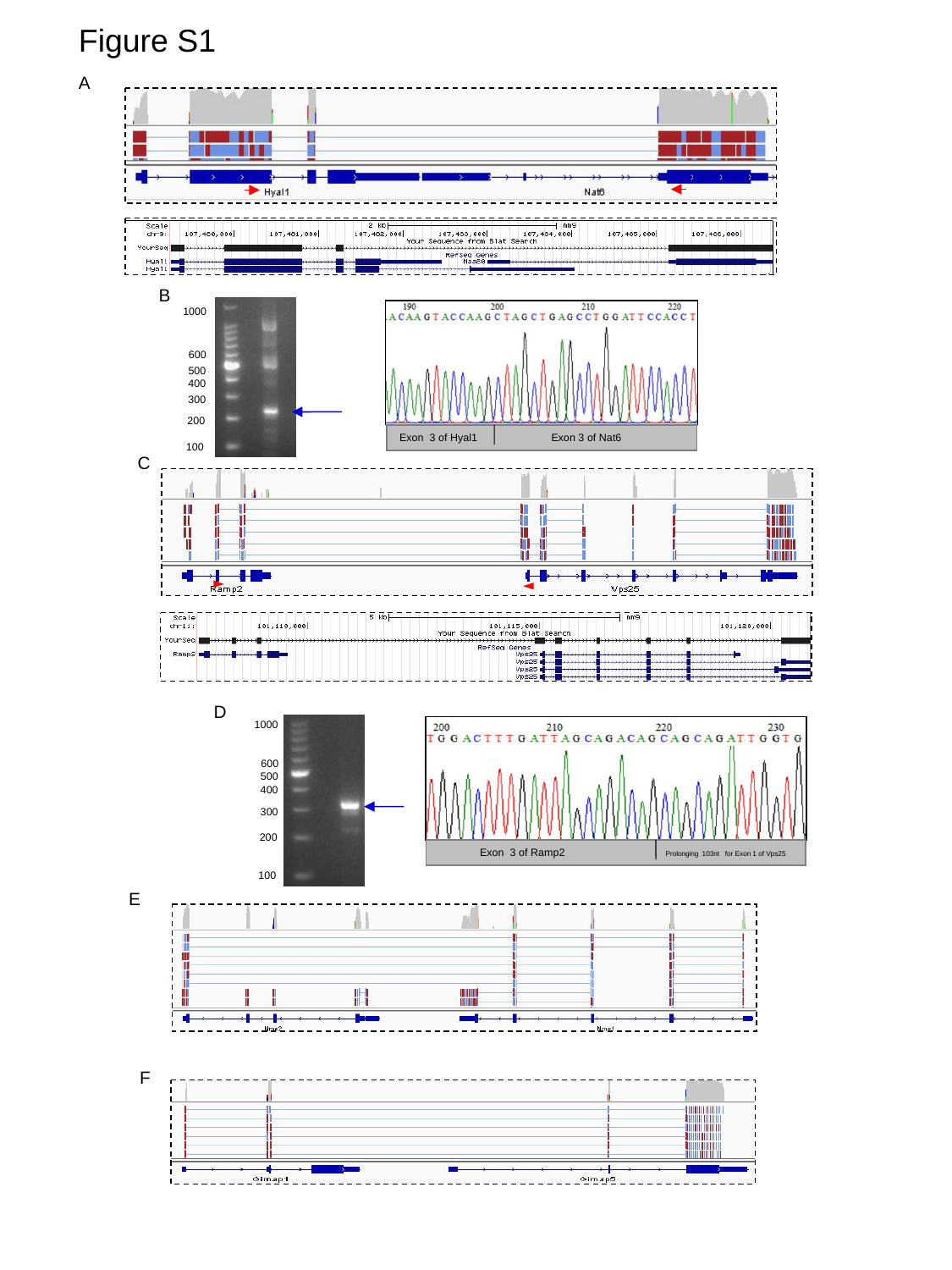

Figure S1
A
B
1000
600
500
400
300
200
Exon 3 of Hyal1 Exon 3 of Nat6
100
C
D
1000
600
500
400
300
200
 Exon 3 of Ramp2 Prolonging 103nt for Exon 1 of Vps25
100
E
F

## Slide 2
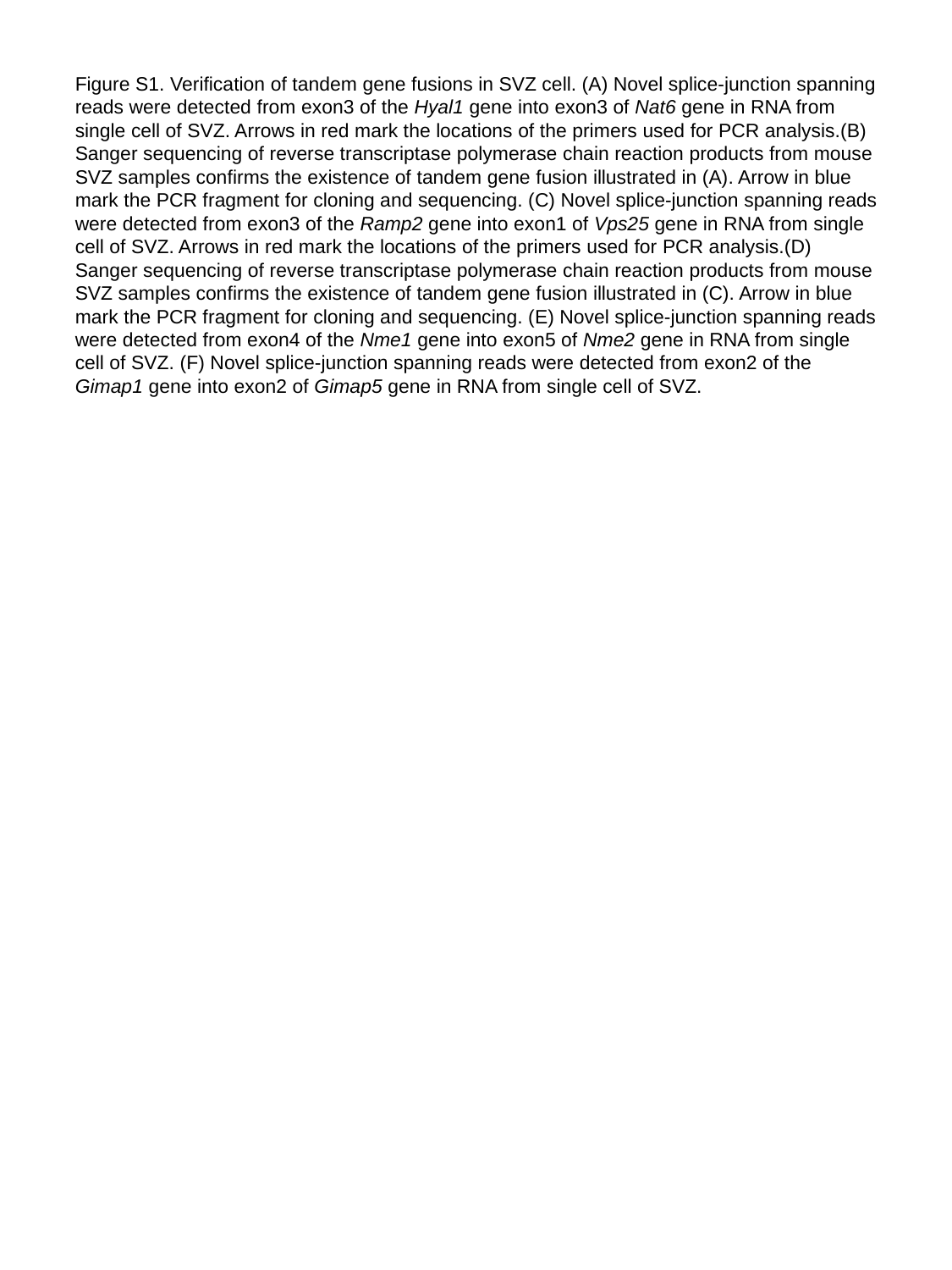

Figure S1. Verification of tandem gene fusions in SVZ cell. (A) Novel splice-junction spanning reads were detected from exon3 of the Hyal1 gene into exon3 of Nat6 gene in RNA from single cell of SVZ. Arrows in red mark the locations of the primers used for PCR analysis.(B) Sanger sequencing of reverse transcriptase polymerase chain reaction products from mouse SVZ samples confirms the existence of tandem gene fusion illustrated in (A). Arrow in blue mark the PCR fragment for cloning and sequencing. (C) Novel splice-junction spanning reads were detected from exon3 of the Ramp2 gene into exon1 of Vps25 gene in RNA from single cell of SVZ. Arrows in red mark the locations of the primers used for PCR analysis.(D) Sanger sequencing of reverse transcriptase polymerase chain reaction products from mouse SVZ samples confirms the existence of tandem gene fusion illustrated in (C). Arrow in blue mark the PCR fragment for cloning and sequencing. (E) Novel splice-junction spanning reads were detected from exon4 of the Nme1 gene into exon5 of Nme2 gene in RNA from single cell of SVZ. (F) Novel splice-junction spanning reads were detected from exon2 of the Gimap1 gene into exon2 of Gimap5 gene in RNA from single cell of SVZ.

## Slide 3
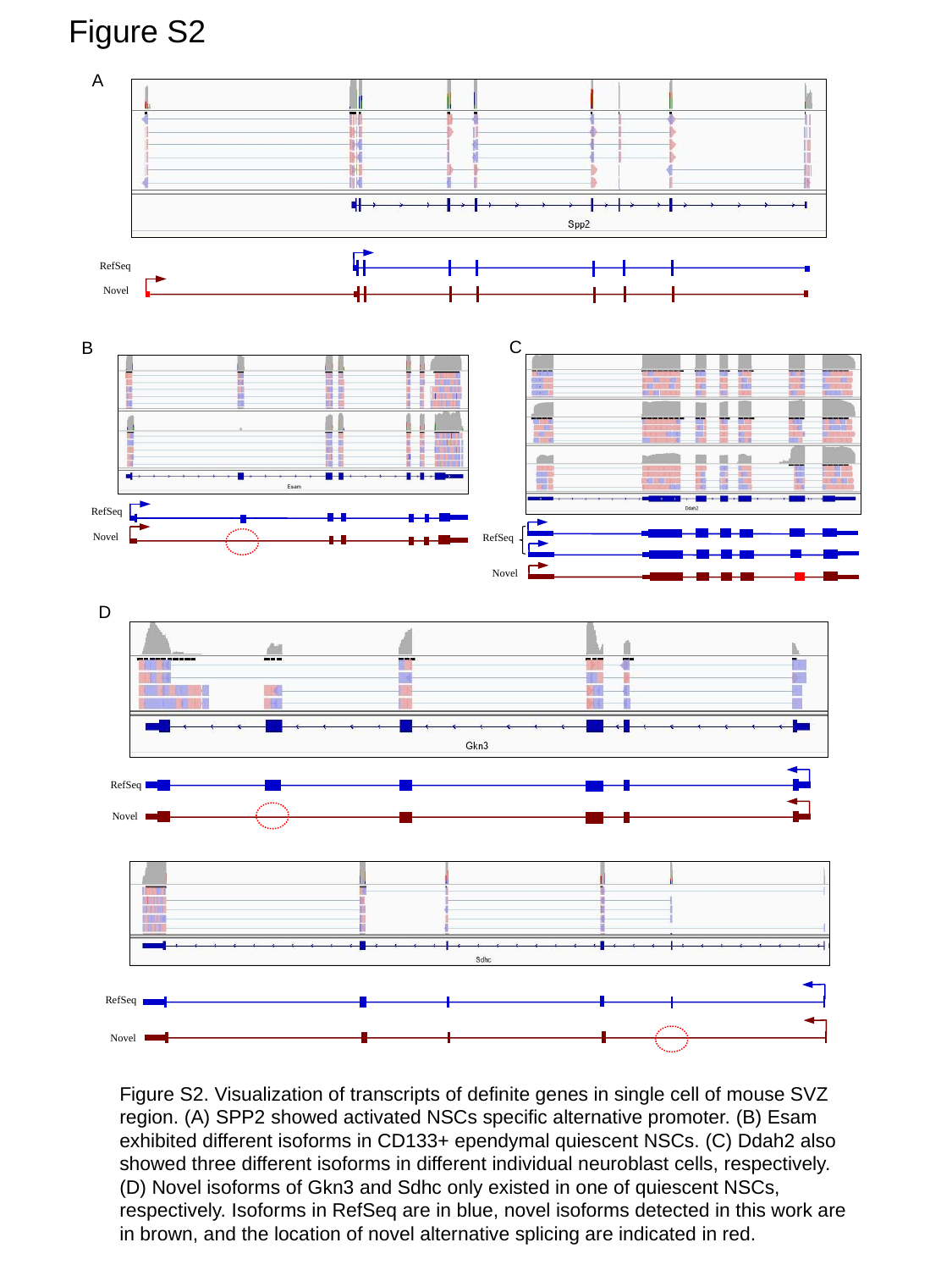

Figure S2
A
RefSeq
Novel
C
B
RefSeq
Novel
RefSeq
Novel
D
RefSeq
Novel
RefSeq
Novel
Figure S2. Visualization of transcripts of definite genes in single cell of mouse SVZ region. (A) SPP2 showed activated NSCs specific alternative promoter. (B) Esam exhibited different isoforms in CD133+ ependymal quiescent NSCs. (C) Ddah2 also showed three different isoforms in different individual neuroblast cells, respectively. (D) Novel isoforms of Gkn3 and Sdhc only existed in one of quiescent NSCs, respectively. Isoforms in RefSeq are in blue, novel isoforms detected in this work are in brown, and the location of novel alternative splicing are indicated in red.

## Slide 4
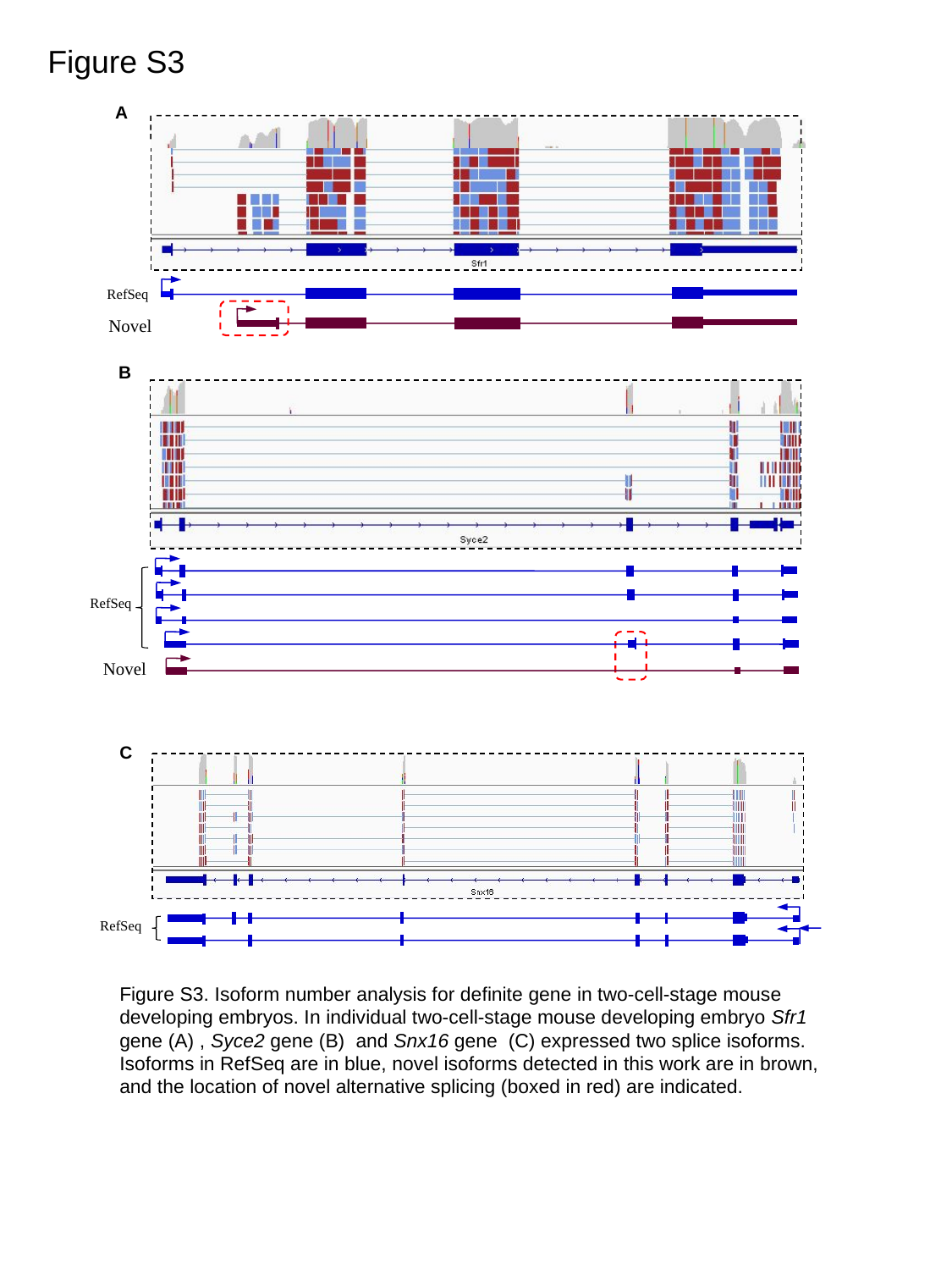

Figure S3
A
RefSeq
Novel
B
RefSeq
Novel
C
RefSeq
Figure S3. Isoform number analysis for definite gene in two-cell-stage mouse developing embryos. In individual two-cell-stage mouse developing embryo Sfr1 gene (A) , Syce2 gene (B) and Snx16 gene (C) expressed two splice isoforms. Isoforms in RefSeq are in blue, novel isoforms detected in this work are in brown, and the location of novel alternative splicing (boxed in red) are indicated.
